# Metabolic disturbances connecting obesity and depression

**DOI:** 10.3389/fnins.2013.00177

**Published:** 2013-10-07

**Authors:** Cecile Hryhorczuk, Sandeep Sharma, Stephanie E. Fulton

**Affiliations:** Department of Nutrition, Faculty of Medicine, CRCHUM and Montreal Diabetes Research Center, Université de MontréalMontreal, QC, Canada

**Keywords:** mood disorders, abdominal obesity, saturated fatty acids, leptin, insulin, adiponectin, resistin, inflammation

## Abstract

Obesity markedly increases the odds of developing depression. Depressed mood not only impairs motivation, quality of life and overall functioning but also increases the risks of obesity complications. Abdominal obesity is a better predictor of depression and anxiety risk than overall adipose mass. A growing amount of research suggests that metabolic abnormalities stemming from central obesity that lead to metabolic disease may also be responsible for the increased incidence of depression in obesity. As reviewed here, a higher mass of dysfunctional adipose tissue is associated with several metabolic disturbances that are either directly or indirectly implicated in the control of emotions and mood. To better comprehend the development of depression in obesity, this review pulls together select findings addressing the link between adiposity, diet and negative emotional states and discusses the evidence that alterations in glucocorticoids, adipose-derived hormones, insulin and inflammatory signaling that are characteristic of central obesity may be involved.

## Introduction

Consistent with its broad impact on physiology and health, obesity is increasingly linked to impairments in central nervous system (CNS) function. Mood disorders are now well recognized as significant risks of obesity and related metabolic illnesses. Obese individuals have about a 55% increased odds of developing depression (Luppino et al., 2010) whereas diabetes is estimated to double the incidence of depression (Anderson et al., 2001). Depression is among the primary causes of disability and its relentless impact can contribute substantially to the burden of metabolic disorders. Beyond diminishing quality of life and functioning, depressed mood presents additional threats to obese individuals by counteracting adherence to treatment and lifestyle changes and increasing the risk of complications. Abdominal adiposity and poor diet quality have been implicated in the development of depressed mood during obesity (Roberts et al., 2003; Dong et al., 2004; Simon et al., 2006; Zhao et al., 2011; Hamer et al., 2012). There is also a bidirectional association between obesity and depression such that depressed individuals are more likely to gain excessive weight due to poor food choices and reduced physical activity (Luppino et al., 2010; Pan et al., 2012). Recent work is beginning to illuminate the biological mechanisms that promote depressed mood in obesity. This review focuses specifically on the impact of adiposity, diet and associated metabolic signals in the development of depression and negative emotional states. First, select findings on the influence of palatable foods, obesity, visceral fat accumulation and dietary fats on emotional states and depression are presented. With an aim to shed light on potential mechanisms, we then review the link between depressive symptomology and some adipose-related metabolic signals, namely glucocorticoids (GCs), leptin, adiponectin, resistin, insulin and inflammatory signals. There are excellent and highly pertinent reviews on the neurobiology of depression (Nemeroff and Vale, 2005; Krishnan and Nestler, 2008; Willner et al., 2012; Sun et al., 2013) and so this subject will not be covered here, rather the potential role of metabolic signals are discussed with regards to what is known about their central actions to affect anxiety, depressive-like behavior and mood.

## Mood, food, and adiposity

Emotions such as joy, frustration and fear can profoundly affect appetite and food choice. In turn, eating palatable foods can modify emotional states by producing feelings of comfort, gratification or disgust which can strongly influence our approach or avoidance of such foods in the future. Positive emotional reactions (i.e., rewarding and hedonic effects) to food are considered to play a major role in overeating and the development of obesity (Fulton, 2010). External or psychological stressors can have divergent effects on feeding behavior such that some individuals increase food intake in response to a stressful experience while others eat less (Oliver and Wardle, 1999; Gibson, 2006; Dallman, 2010). Similarly, conditions of chronic stress can lead to diminished appetite and weight loss in some individuals whereas it can have an opposite effect in others by stimulating consumption of palatable and rewarding foods that blunt stress responses (Adam and Epel, 2007). For example, a recent study found that women reporting higher chronic stress with low cortisol reactivity to an acute social stress test consumed more calories from chocolate cake and less vegetables than those experiencing low chronic stress (Tryon et al., 2013). Likewise, a similar positive correlation between social stress and choice of palatable, energy rich foods over fruits and vegetables is observed in children (Cartwright et al., 2003). Stress-induced preference for palatable food has been documented in several human (Stone and Brownell, 1994; Epel et al., 2004) and animal studies (Dallman et al., 2003, 2005; Cottone et al., 2009) while other work demonstrates the effects of such foods to dampen signs of stress and anxiety following exposure to a stressor or conditions of chronic unpredictable stress (Pecoraro et al., 2004; la Fleur et al., 2005; Maniam and Morris, 2010; Ulrich-Lai et al., 2010; Finger et al., 2011, 2012). There is a strong connection between the consumption of high-fat and high-sugar foods and positive emotions, and despite the divergence in eating behavior, an overall increase in tasty, energy-rich foods is reported, independent of stress-induced hyperphagia or hypophagia (Gibson, 2006; Dallman, 2010).

Mood states such as anxiety and depression can also affect food choice and energy metabolism. Individuals experiencing depressed mood show preference for and consumption of palatable “comfort foods” and self-report use of these foods as means to alleviate negative feelings (Macht, 2008). While short-term consumption of palatable foods can provide relief from negative emotions and mood states, chronic consumption of calorically-rich foods and subsequent increases in adiposity may promote vulnerability to depression and anxiety (Novick et al., 2005; Simon et al., 2006; Kloiber et al., 2007; Sharma and Fulton, 2013). In a recent study, we found that mice consuming a saturated high-fat diet (HFD) for 12 weeks show depressive-like features characterized by greater immobility in the forced swim task and reduced exploratory behavior in elevated plus maze and open field tests (Sharma and Fulton, 2013). HFD-induced behavioral changes were accompanied by elevated basal hypothalamic-pituitary-adrenal (HPA) activity and reactivity in response to stress. Separate findings suggest that negative emotional states and increased stress sensitivity caused by prolonged high-fat feeding do not rely on severe diet-induced obesity (DIO): six weeks of saturated HFD, leading to a more moderate gain in weight (~11% relative to low-fat diet controls) and without influencing basal corticosterone levels, produced sucrose anhedonia (reduced ability to experience pleasure/reward), anxiety-like behavior and heightened stress-induced HPA activation (Sharma et al., 2012). Furthermore, when high-fat fed mice were returned to a normal chow diet craving for sucrose and high-fat food and behavioral and biochemical signs of anxiety were potentiated (Sharma et al., 2012). Several groups have shown that removal of a high-fat or high-sugar diet following prolonged intermittent or regular intake can increase behavioral and physiological signs of depression and anxiety (Avena et al., 2008a; Teegarden et al., 2008; Cottone et al., 2009; Iemolo et al., 2012; Sharma et al., 2012) or withdrawal (Avena et al., 2008b; Pickering et al., 2009; Sharma et al., 2012). Results of a new study suggest endocannabinoid signaling in the central nucleus of the amygdala is increased as a means to counteract increased anxiety elicited by palatable food withdrawal (Blasio et al., 2013). Thus, not only can chronic high-fat feeding promote negative emotional states but it may also enhance sensitivity to stress, including that triggered by dieting, to perpetuate a vicious cycle of overeating, weight gain and depressed mood.

Evidence suggests that diets high in saturated fat and relatively low in polyunsaturated and monounsaturated fatty acids contribute to the pathogenesis of both mood and metabolic disorders during obesity. The consumption of foods rich in saturated and/or trans-fat, like the Western diet, is associated with an increased incidence of depression whereas diets containing mostly unsaturated fats, such as the Mediterranean diet, appear to reduce the odds of depression (Sanchez-Villegas and Martinez-Gonzalez, 2013). Other reports indicate that inadequate dietary polyunsaturated fatty acids (PUFA) are associated with a higher incidence of depression (Peet et al., 1998) and that increasing omega-3 PUFA by greater consumption of fish can either decrease depressive symptoms in humans (Lin and Su, 2007; Sanchez-Villegas et al., 2007; Oddy et al., 2011; Park et al., 2012a) and rodents (Moranis et al., 2012; Park et al., 2012b) or have no effect on mood (Ruusunen et al., 2011). A considerable amount of data shows that diets rich in saturated fatty acids are associated with increases in overall adiposity and bias fat accumulation in abdominal stores. As compared to individuals on a Mediterranean diet, those consuming a diet high in saturated fat have increased weight gain, a greater volume of visceral adipose tissue, larger waist circumference and more cardiovascular disease mortality (Schulze et al., 2006; Molenaar et al., 2009; Romaguera et al., 2009, 2010; Mozaffarian et al., 2011; Estruch et al., 2013; Nazare et al., 2013). The accumulation of adipose tissue in abdominal stores is more important than the total amount of body fat in predicting the risk of several complications of obesity including insulin resistance and the metabolic syndrome (cluster of metabolic abnormalities and cardiovascular risk factors with impaired insulin sensitivity) (Despres and Lemieux, 2006; Tchernof and Despres, 2013) whereas the Mediterranean diet has been shown to be largely protective against these metabolic risks (Riccardi and Rivellese, 2000). The higher mass of dysfunctional adipose tissue in obesity may not only cause or exacerbate metabolic abnormalities that give rise to metabolic disease but also neurobiological impairments that give rise to mood disorders. Indeed, central adiposity is a better predictor of depression and anxiety risk than body weight or body mass index (BMI) (Weber-Hamann et al., 2002; van Reedt Dortland et al., 2013a). These findings suggest that excessive saturated fat intake and metabolic alterations that produce or arise from high abdominal fat mass may serve as common causal elements for both metabolic and mood disorders.

Apart from stimulating visceral fat accumulation, saturated fat intake increases circulating concentrations of fatty acids like palmitate which can enter the brain to have deleterious effects on neural functions. Indeed, palmitate has been shown to impair leptin and insulin receptor signal transduction in the hypothalamus to thereby reduce their catabolic actions and promote weight gain (Benoit et al., 2009; Kleinridders et al., 2009). As described below, leptin and insulin are implicated in enhanced mood and thus it is conceivable that reduced responses to these hormones elicited by saturated high fat feeding could contribute to negative emotional states. Supporting the possibility that high CNS palmitate levels modulate neural mechanisms controlling emotions, a recent study demonstrates that serum concentrations of palmitate correlated positively with depression in humans (Tsuboi et al., 2013). In addition, we recently found that male adult Wistar rats consuming a palm oil HFD for 8 weeks and with higher serum levels of palmitate show greater anxiety-like behavior in an open field test relative to rats consuming an isocaloric olive oil HFD (Hryhorczuk et al., unpublished). Interestingly, these effects were not accompanied by changes in body weight, glycemia and plasma leptin and insulin levels suggesting that the anxiogenic phenotype could be related to the effects of saturated fats to stimulate HPA disturbances and/or inflammation. As elaborated in the next section, visceral fat deposition and dyslipidemia are associated with several endocrine and metabolic changes that have been implicated in the CNS control of emotional states and mood.

## Metabolic signals and emotional states

**Figure 1:**
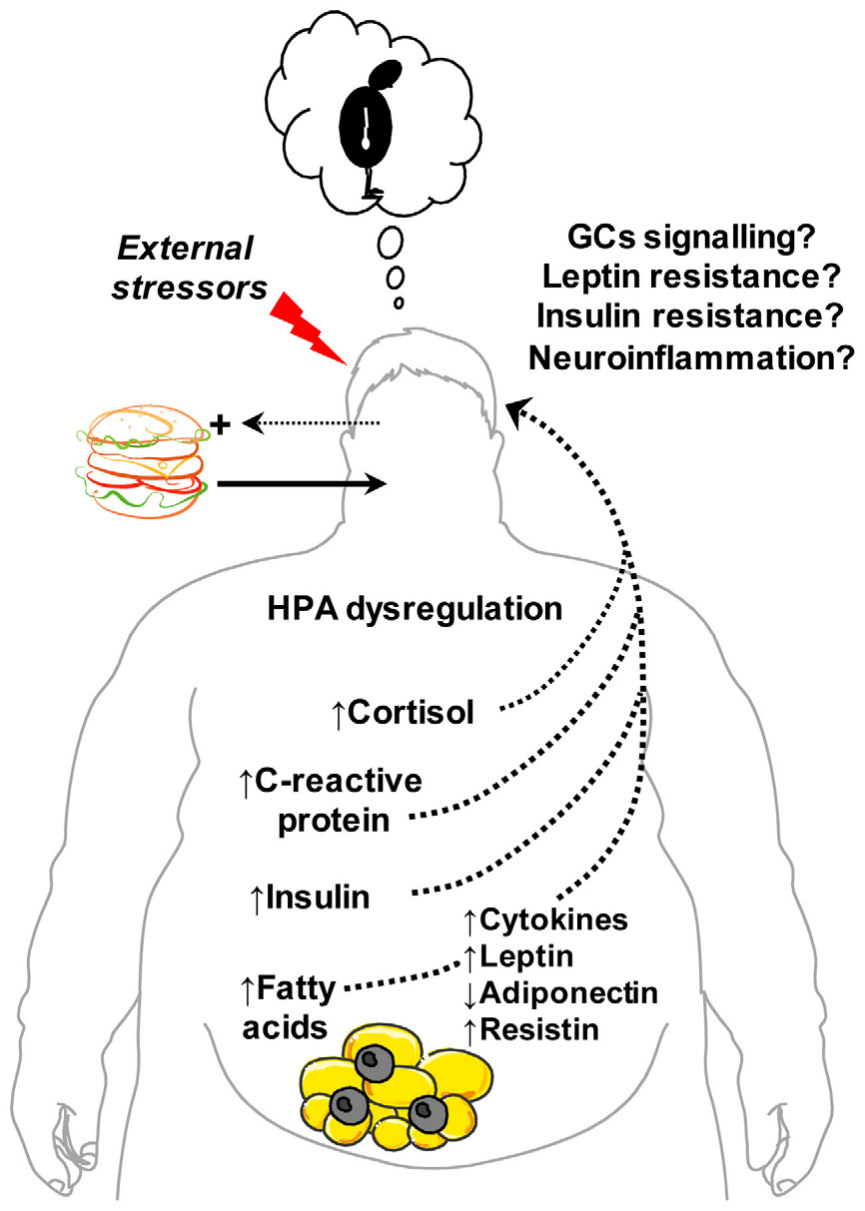
**Metabolic signals and disturbances linking obesity and abdominal adiposity with depression.** Central obesity and related metabolic changes correlate positively with depression. Excessive intake of foods high in saturated fat promotes weight gain, visceral fat accumulation and increased risk of depressed mood. Endocrine changes associated with abdominal obesity include hypothalamic-pituitary-adrenal (HPA) dysregulation and altered plasma levels of cortisol, leptin, adiponectin, resistin and insulin—hormones implicated in the central control of emotion and mood. Obesity-induced impairments in brain glucocorticoids (GCs), leptin and insulin receptor signaling are posited to link hypercortisolemia and leptin and insulin resistance to depression. Central fat accumulation also stimulates the release of inflammatory cytokines (e.g., tumour necrosis factor-α, interleukin-1β) and signals (C-reactive protein) that can promote neuroinflammatory responses and depressive behavior. Increased entry of saturated free fatty acids into the brain may also propagate neuroinflammation, leptin/insulin resistance and, consequently, depression. In parallel, increased vulnerability to external stressors, negative emotional states and adverse cognitive style (e.g., poor self-image) associated with overweight and obesity can potentiate consumption of energy-dense “comfort” foods to fuel a vicious cycle of central obesity, metabolic dysfunction and depression. This figure was produced using *Servier Medical Art*.

A meta-analysis of longitudinal data figures obesity (BMI ≥ 30) to increase the overall risk of onset of depression by 55% in Americans while overweight (BMI 25–29.9) to heighten the incidence of depression by 27% (Luppino et al., 2010). Despite the comorbidity of obesity and depression, it is important to note that obese individuals classified as “metabolically healthy,” that is without associated cardiometabolic risk factors (high blood pressure, reduced high-density lipoprotein cholesterol and increased triglycerides, glycated haemoglobin and C-reactive protein), do not appear to have a heightened risk for depression (Hamer et al., 2012). Accordingly, an increasing amount of data points toward unhealthy diet, visceral adiposity and associated metabolic changes as culprits in obesity-induced depression (Roberts et al., 2003; Dong et al., 2004; Simon et al., 2006). As discussed here and summarized in Figure 1, several endocrine and metabolic abnormalities have been linked to depressed mood or depressive-like behavior including hypercortisolemia, insulin and leptin resistance and metabolic inflammatory signals. These signals are positively associated with central fat accumulation and thus are important candidate molecules tying obesity to depression.

### Glucocorticoids

HPA axis dysfunction and elevated GCs are implicated in the pathophysiology of both obesity and depression. GCs exert pleiotropic effects on metabolic, endocrine, immune and behavioral functions. Under normal physiological conditions of acute stress, the HPA axis is activated to release GCs which have an adaptive value to restore energy balance by increasing insulin, motivation for palatable food (Piazza and Le Moal, 1997; Dallman et al., 2006; Dallman, 2010) and mobilizing stored energy and directing it to central stores (Mann and Thakore, 1999). The critical actions of GCs on adipose tissue deposition were demonstrated by early studies showing that adrenalectomy prevents obesity in Zucker rats and this is reversed by replacement of corticosterone (Freedman et al., 1985; Castonguay et al., 1986). Fat mass is associated with increased cortisol rise at the time of waking and high cortisol reactivity under stressful situations (Therrien et al., 2007, 2008; Mujica-Parodi et al., 2009). But while cortisol production and turnover are increased in obesity, overall systemic cortisol levels have been shown to be normal or low in obesity (Hautanen et al., 1997). Rather, dysregulation of HPA axis activity is more a function of body fat distribution rather than total fat mass as abnormal diurnal variation in circulating cortisol levels is positively associated with the waist-to-hip ratio (Lasikiewicz et al., 2008). Over-activation of the HPA axis in subjects with abdominal obesity is suggested by elevated response to corticotrophin releasing hormone (CRH) stimulation, and increased basal and stimulated response to stress (Pasquali, 2012). Excess systemic cortisol has been shown to result in a 2- to 5-fold increase in visceral adipose tissue (Brown et al., 2004), and as such individuals under chronic stress are likely to have more visceral fat (Adam and Epel, 2007; Kyrou and Tsigos, 2009).

Studies have found small elevations in serum cortisol concentrations associated with depression (Parker et al., 2003; Raison and Miller, 2003; Stetler and Miller, 2011). Further, individuals with Cushing's syndrome (characterized by cortisol hypersecretion and abdominal obesity) exhibit behavioral and neurobiological features of depression (Sonino et al., 1998). GCs not only act peripherally to maintain energy homeostasis but also feedback to the CNS to modulate HPA activity and the emotional and behavioral effects of stress (Herman et al., 2003). GCs are known to target GC receptors [including mineralocorticoid (MC) receptors] in midbrain and limbic circuits that regulate reward and emotional processes (Arnett et al., 2011; Solomon et al., 2012; Wang et al., 2013). Repeated administration of corticosterone to rodents is reported to produce depressive-like behavior (Kalynchuk et al., 2004; Gregus et al., 2005) whereas GC receptor overexpression in the forebrain increases depressive-like behavior in the forced swim test and anxiety-like behavior in the elevated plus maze task (Wei et al., 2004). In contrast, loss of forebrain GC receptors has also been shown to increase depressive-like behavior in the forced swim and tail suspension tasks (Boyle et al., 2005) and increase stress-induced locomotor activation (Boyle et al., 2006). Finally, suggesting a particular role for MC receptor signaling in anxiety, MC receptor overexpression in the forebrain (Rozeboom et al., 2007) or specifically in basolateral amygdala (Mitra et al., 2009) reduces anxiety-like behavior. These contradictory results may be reconciled by findings that increasing forebrain GR receptor signaling enhances the magnitude of both positive and negative emotional responses depending upon the test employed suggesting that GC are important for “emotional lability” (Wei et al., 2004). On the whole, however, many findings support a pro-depressive effect of GC surplus, especially in the context of their strong influence to inhibit brain development and growth. Excess GCs promote hippocampal atrophy and decrease neurogenesis in the hippocampus—actions that have been well-implicated in the effect of GC on depressive behavior (McEwen, 1997; Bremner, 2006; Goebel et al., 2010; Schoenfeld and Gould, 2012).

It is important to note that hypercortisolism is often associated with melancholic depression, a subtype of clinical depression that is accompanied by anhedonia, hypophagia and weight loss; while atypical depression, the most common form of depression, is characterized by reduced HPA activity, improved mood in response to positive events and increased appetite, carbohydrate craving and frequently weight gain (Juruena and Cleare, 2007). Despite these categorizations, there is evidence of an association between hypercortisolemic depression and abdominal fat accumulation (Weber-Hamann et al., 2002). Hypercortisolemia in depressed individuals is accompanied by decreased GC-mediated negative feedback and increased release of CRH from the paraventricular nucleus (PVN) (Holsboer, 2000). Along these lines, we found that the increased anxiety-like behavior of the rats subjected to the saturated palm oil HFD was accompanied by elevated basal corticosterone levels and reduced GC-mediated negative feedback (dexamethasone suppression test) relative to rats consuming the calorically-matched olive oil HFD (Hryhorczuk et al., unpublished). As no differences in weight, leptin, insulin and blood glucose were detected, we hypothesize that increased corticosterone and/or reduced GC receptor signaling in limbic areas may in part mediate the effects of a saturated HFD to elicit negative emotional states. It is interesting to speculate that GC surplus in obesity may be associated with central impairments in the intracellular metabolism of GCs by 11β-hydroxysteroid dehydrogenases (11β-HSDs), enzymes that are well-implicated in visceral fat accumulation and affiliated peripheral metabolic abnormalities such as insulin resistance (Tchernof and Despres, 2013). 11βHSDs are widely expressed in the brain, including the hippocampus and amygdala, and are linked to changes in emotional and cognitive functions (Wyrwoll et al., 2011). It is clear that HPA abnormalities serve as common elements in the pathophysiology of both obesity and depression, but their direct contribution to emotional disturbances elicited by DIO have not been established. Notably, the data collectively suggest that diets rich in saturated fat and visceral fat accumulation, but not body mass *per se*, are important mediators of HPA disturbances and depressive symptomology associated with obesity.

### Adipose-derived hormones

#### Leptin

As an endocrine organ, adipose tissue secretes numerous peptide hormones that target the brain and other tissues to regulate metabolism and behavior. Leptin is an adipokine that circulates in proportion to the size of the fat mass (Maffei et al., 1995). Apart from its numerous actions on physiological processes such as appetite, energy expenditure and neuroendocrine function, leptin is linked to human depression and has been shown to have antidepressant and anxiolytic effects in rodents (Asakawa et al., 2003; Liu et al., 2010b; Yamada et al., 2011). Epidemiological findings on leptin levels and depression are conflicting. Several studies find that individuals with major depressive disorder (MDD) have lower plasma leptin levels compared to healthy controls with similar BMI (Kraus et al., 2001; Atmaca et al., 2002, 2008; Westling et al., 2004; Jow et al., 2006). However, other reports show that plasma leptin levels are increased in women with depressive disorder (Rubin et al., 2002; Esel et al., 2005; Zeman et al., 2009) and that leptin levels are either increased (Kraus et al., 2002; Esel et al., 2005; Schilling et al., 2013) or not changed (Kraus et al., 2002; Schilling et al., 2013) by antidepressant treatment. In depressed individuals suffering from loss of appetite plasma leptin concentrations were reported not to differ from those of healthy controls (Deuschle et al., 1996) whereas another study found higher serum leptin only in atypical depressive patients in which increased appetite is often observed (Gecici et al., 2005). In older men, the combination of elevated visceral fat and high leptin levels was associated with depression onset (Milaneschi et al., 2012). Rodent studies, on the other hand, present more conclusive findings. Lack of leptin (obese *ob/ob* mice) or its receptor (obese *db/db* mice) is associated with increased behavioral despair in the forced swim task (Collin et al., 2000; Sharma et al., 2010; Yamada et al., 2011). Repeated leptin treatment has also been shown to reduce anxiety in *ob/ob* mice (Asakawa et al., 2003). Systemic or central leptin administration in normal mice has antidepressant effects as measured by the tail suspension test, forced swim task and social interaction test and has anxiolytic effects in the elevated plus maze (Liu et al., 2010a; Yamada et al., 2011). In sum, a greater part of the human research ties low leptin levels to depression but there is discordancy amongst these investigations which may be a function of the subtype of depression, the age and sex of participants and perhaps, as elaborated below, the onset of leptin insensitivity generated by high leptin levels.

One of the ways in which leptin may affect emotions is via its influence on HPA activity. A negative correlation between basal concentrations of leptin and mobilization of cortisol is reported in humans (Komorowski et al., 2000). Leptin deficient *ob/ob* mice have elevated corticosterone which is reduced by leptin replacement (Garthwaite et al., 1980; Arvaniti et al., 2001). Systemic leptin administration lowers corticosterone levels and prevents the induction of CRH synthesis in the PVN and the activation of CRH neurons observed in food-deprived *ob/ob* mice (Huang et al., 1998). In turn, chronic unpredictable mild stress in rats which leads to depressive-like behaviors activates the HPA axis and decreases serum leptin levels (Ge et al., 2013). Beyond modulating HPA activity, leptin also targets the long-form of its receptor (LepRb) in midbrain and forebrain loci to affect emotional processes. Leptin has antidepressant effects when administered into the hippocampus, an area mediating cognitive impairments of depression whereas genetic deletion of hippocampal LepRb results in a depression-like phenotype (Asakawa et al., 2003; Lu et al., 2006; Finger et al., 2010; Liu et al., 2010a; Guo et al., 2013). Loss of LepRb specifically in glutamatergic neurons of the forebrain (mainly hippocampus and prefrontal cortex) was recently reported to elicit depressive-like behavior without affecting anxiety (Guo et al., 2012). Leptin also functionally activates dopamine neurons in the ventral tegmental area (VTA) of the midbrain where it reduces dopamine neuronal firing while increasing dopamine availability (Fulton et al., 2006; Hommel et al., 2006). Selective deletion of LepRb from midbrain dopamine neurons has been shown to increase anxiety-like behavior, but not depressive-like behavior, in several behavioral tests via increased D1 receptor signaling in the central nucleus of the amygdala (Liu et al., 2011). Finally, recent work shows that leptin levels are positively associated with stress-induced dopamine release (Burghardt et al., 2012). Collectively, the data demonstrates that the antidepressant actions of leptin are mediated by LepRb signaling in limbic and prefrontal nuclei whereas leptin action in dopamine neurons of the ventral midbrain that innervate the central nucleus of the amygdala underlie the anxiolytic actions of leptin.

Leptin levels are associated with risk of depression onset in men with a significant amount of visceral fat (Milaneschi et al., 2012) and correlate positively with depressive symptoms in patients with type 2 diabetes (Labad et al., 2012). In conditions of obesity, and particularly central obesity that favors insulin resistance and type 2 diabetes, leptin sensitivity is diminished owing to lower CNS leptin entry (decreased CSF: plasma leptin ratio) and defective LepRb signaling (Myers et al., 2012). This phenomenon known as leptin resistance is characteristic of obesity and may explain how the risk of mood disorders is elevated in obese states associated with high circulating leptin levels. Compatible with this notion, mice rendered obese by a HFD show reduced sensitivity to the antidepressant actions of leptin and to the effects of leptin to increase hippocampal brain-derived neurotrophic factor (BDNF) concentrations (protective against depression) relative to low-fat diet controls (Yamada et al., 2011). Further, leptin insensitivity may exacerbate HPA dysregulation in obesity (Collura et al., 2009) and thereby enhance the mass of dysfunctional central adipose stores in a cortisol-dependent manner. Features of leptin resistance have been reported in the midbrain VTA where mesolimbic DA neurons reside (Matheny et al., 2011), although it is not yet known whether or not reduced leptin sensitivity in this region has anxiogenic effects as one would predict. Leptin resistance would appear to affect multiple neural and endocrine pathways that affect emotions and mood including hippocampal and mesolimbic dopamine pathways and HPA activity, and thus represents another strong candidate mechanism underlying depression in obesity.

#### Adiponectin

Adiponectin is another adipose-derived hormone that is well implicated in energy homeostasis and insulin resistance (Turer and Scherer, 2012) and that has more recently been tied to depression. While plasma adiponectin is found at high concentrations in healthy subjects it often correlates negatively with obesity, waist circumference and visceral fat in humans (Arita et al., 1999; Cnop et al., 2003; Ryo et al., 2004; Hanley et al., 2007; Cohen et al., 2011; Mente et al., 2013) and rodents (Maeda et al., 2001; Milan et al., 2002; Delporte et al., 2004; Ye et al., 2007). However, metabolically healthy obese subjects have plasma levels of adiponectin similar to lean controls (Aguilar-Salinas et al., 2008; Morrison et al., 2011; Doumatey et al., 2012) suggesting that adiponectin levels changes are secondary to metabolic disturbances found in obesity. In humans, plasma adiponectin levels are reported to negatively correlate with depressive measures in patients with MDD and in women with depressive disorder (Zeman et al., 2009). Alternatively, other studies report a positive relationship between adiponectin levels and depressive and anxiety symptoms (Wilhelm et al., 2013) and men with subsyndromal depression (Jeong et al., 2012); or no change in patients with MDD (Jeong et al., 2012). Antidepressant therapy in individuals with MDD does not appear to affect adiponectin and resistin levels (Lehto et al., 2010). Recent work shows that chronic social defeat in mice decreases the level of plasma adiponectin and that recapitulating low levels of adiponectin by using haploinsufficient mice (Adipo±) increases the incidence of stress-induced depressive-like behaviors due to impaired HPA axis negative feedback (Liu et al., 2012). Moreover, it was found that, like leptin, central administration of adiponectin has antidepressant effects as assessed in the forced swim test and tail suspension test (Liu et al., 2012). In summary, while animal studies suggest an antidepressant action of adiponectin, the link between plasma adiponectin concentrations and depression in humans is less clear which may be connected to the type of depressive disorder and the effects of sex and antidepressant treatment.

#### Resistin

Adipocyte-derived resistin is linked to insulin resistance in rodent models, however, human resistin is released from macrophages and its association with cardiometabolic disease is less defined (Schwartz and Lazar, 2011). Plasma resistin levels in relation to obesity are not as clear as those for adiponectin. Early studies found that circulating resistin levels are elevated in genetic and DIO in mice (Steppan et al., 2001) and obese humans (Degawa-Yamauchi et al., 2003; Owecki et al., 2011; Sadashiv et al., 2012) whereas other studies found that resistin is downregulated in human obesity (Way et al., 2001; Ye et al., 2013) and rodent obesity (Milan et al., 2002; Maebuchi et al., 2003). As for the relationship between resistin and depression in humans, resistin levels were found to be positively correlated with atypical but not typical depression (Lehto et al., 2010). In addition, human resistin levels were positively linked with salivary cortisol in depressed patients (Weber-Hamann et al., 2007) which is in line with a study reporting higher plasma resistin levels in women suffering from Cushing's syndrome (Krsek et al., 2004). Conversely, resistin levels are lower in patients receiving antidepressant treatment who have remitted from depression (Weber-Hamann et al., 2007). As it currently stands, there are no documented reports exploring the impact of resistin treatment or its loss of function in animal studies of anxiety and depression. The epidemiological data would predict resistin treatment to have pro-depressant actions, a potential finding that could be related to resistin's status as an inflammatory marker.

### Insulin

Depression is at least twice as common among those with diabetes and is associated with a more unfavorable prognosis (Anderson et al., 2001). Depression often appears at the prediabetes stage which is marked by insulin resistance (Kan et al., 2013). Individuals with insulin resistance that are overweight or obese (Platt et al., 2013), with metabolic syndrome (Koponen et al., 2008; Akbaraly et al., 2009; Almeida et al., 2009; Pulkki-Raback et al., 2009) or with abdominal obesity and metabolic syndrome (Hamer et al., 2012) are more likely to develop depression. However, some literature points toward little or no association between metabolic syndrome/insulin resistance and depression (Adriaanse et al., 2006; Platt et al., 2013; Shen and Bergquist-Beringer, 2013). A recent systematic review and meta-analysis found a small but significant cross-sectional association between depression and insulin resistance (Kan et al., 2013). Another recent study found that treating patients with MDD and abdominal obesity or metabolic syndrome with the insulin-sensitizing drug pioglitazone for 12 weeks alone or in combination with an antidepressant therapy reduced signs of depression and anxiety (Kemp et al., 2012). Attenuation of depressive symptoms correlated positively with the reduction in insulin resistance. Similarly, rosiglitazone administered to normal chow-fed mice and rats was reported to decrease immobility time in tail suspension and forced swim tests suggestive of an antidepressant action (Eissa Ahmed et al., 2009). Collectively, the data suggest that insulin resistance may be causal to depressed mood during obesity while increasing sensitivity to insulin has antidepressant actions.

Several studies have sought to determine the role of CNS insulin in the control of emotional states. Third ventricle injection of an insulin receptor antisense RNA in rats was reported to stimulate depressive-like behavior by increasing immobility in forced swim test and decreasing open arm entries in elevated plus maze (Grillo et al., 2011). Intranasal insulin treatment provides a means to deliver insulin to the brain by bypassing the blood–brain barrier. Daily intranasal insulin treatment for 8 weeks in healthy men was shown to ameliorate self-reported mood (Benedict et al., 2004). Other clinical studies show that intranasal insulin reduces cortisol levels and thus may have antidepressant actions by lessening visceral obesity (Chapman et al., 2013). Intranasal insulin delivery in mice for 7 days was shown to have anxiolytic properties in the light/dark box test, but insulin did not have any effect on anxiety behavior in the marble-burying test and elevated plus maze. In prediabetic (hyperglycaemic and hyperinsulinemic) mice fed for 55 weeks with a 30% kcal fat diet, the anxiolytic effects of intranasal insulin are somewhat diminished (Marks et al., 2009): intranasal insulin failed to reverse the anxiogenic effects of DIO in the light/dark box test while it attenuated anxiety in the elevated plus maze and the marble-burying test. These data suggest that DIO may provoke increased anxiety through impairments in central insulin signaling. In support of this possibility, chronic intake of HFD elicits impairments in hypothalamic insulin receptor signaling (De Souza et al., 2005) and similar resistance to insulin is documented in other brain areas (Kim and Feldman, 2012). By and large, available evidence suggests that insulin has mood-enhancing effects or point to a positive correlation between insulin resistance and depression. However, it will be important to determine if intranasal insulin has antidepressant effects in depressed individuals and, if so, whether this action is maintained in obesity. In addition, more research is required to identify the brain sites mediating the effects of insulin on mood. One likely site of action is the VTA as recent evidence demonstrates that insulin inhibits excitatory glutamatergic inputs onto dopamine neurons (Labouebe et al., 2013). In view of leptin's actions to diminish behavioral despair and anxiety via hippocampal and ventral midbrain nuclei, respectively, regions where insulin receptors are also expressed, it is interesting to speculate that these sites could be important for an influence of insulin on emotional states.

### Inflammatory signals

Obesity is characterized by a state of prolonged low-grade inflammation and several lines of evidence implicate immune-mediated tissue inflammation as an important element linking obesity to insulin resistance. Likewise, numerous findings implicate immune activation in the pathogenesis of depression (Dunn et al., 2005; Dantzer et al., 2008). Elevated levels of inflammatory signals have been associated with the severity of depression (Levine et al., 1999; Dunn et al., 2005). In addition, cancer patients receiving cytokine treatment are more likely to experience depressive symptoms (Musselman et al., 2001). Cytokines are important mediators of innate and adaptive immunity. Both peripherally- and centrally-derived cytokines can stimulate CNS cytokine receptors and several findings point to cytokines as strong modulators of mood. Administration of lipopolysaccharide generates the release of interleukin 1β (IL1β) and tumour-necrosis factor-alpha (TNFα) in the brain to produce sickness behaviors characterized by social withdrawal, decreased exploratory behavior and depressive-like symptoms (Dunn et al., 2005). Direct administration of IL1β and TNFα (but not IL6) to the brain produces depressive symptomology in rodents (Goshen et al., 2008) whereas central administration of an IL1β receptor antagonist during chronic stress (Koo and Duman, 2008) or targeted deletion of the gene encoding TNFα produces antidepressant like effects (Simen et al., 2006). Similarly, preclinical studies show that blocking pro-inflammatory cytokine signaling can produce an antidepressant effect. An important transcriptional target of IL1β and other cytokines is the inhibitor of nuclear factor kappa B kinase (IκK)/nuclear factor kappa B (NFκB) pathway. Activation of IκK/NFκB specifically in the nucleus accumbens (NAc), a region viewed to mediate hedonic deficits in depression, using viral-mediated gene transfer was shown to increase anxiety- and depressive-like behavior using the openfield and forced swim tests, respectively (Koo et al., 2010). In contrast, inhibition of IκK/NFκB in the NAc decreased depressive-like behavior (Christoffel et al., 2012). Together, these findings suggest that elevated levels of pro-inflammatory cytokines in the brain and NAc IκK/NFκB signaling increases anxiety and depressive symptomology.

Obesity provides a means by which inflammatory responses in the brain can be generated. Consistent with this view, a recent study demonstrates that several gene markers of neuroinflammation are upregulated in the hypothalamus of rodents during high-fat feeding including IL6, TNFα, IKKβ and NFκB (Thaler et al., 2012). This metabolic inflammatory response in the hypothalamus appeared rapidly after the onset of high-fat feeding, waned and then escalated in magnitude and diversity during chronic high-fat feeding. Increased infiltration, activation and proliferation of microglia and astrocytes, called reactive gliosis, was also noted in rodents following 4 weeks of high-fat feeding along with similar postmortem signs of gliosis and neuronal injury in the hypothalami of obese individuals (Thaler et al., 2012). At a functional level, elevated inflammatory conditions in the hypothalamus have been shown to contribute to leptin resistance (Kleinridders et al., 2009), insulin resistance (De Souza et al., 2005) and to neuroendocrine impairments, obesity and glucose intolerance in a manner that depends on IKKβ/NFκB signaling (Zhang et al., 2008).

A recent, large-scale epidemiological study evaluated the impact of HPA activity, autonomic nervous system, inflammation and lifestyle factors on adverse associations of anxiety and depression severity with high and low-density lipoprotein cholesterol, triglycerides, BMI and waist circumference. Interestingly, the inflammatory marker C-reactive protein (CRP) had the most consistent impact explaining 14–53% of the associations of anxiety and depression severity with abdominal obesity and dyslipidemia (van Reedt Dortland et al., 2013b). In a separate study, men with low-grade inflammation, high CRP levels and depressive symptoms were more likely to develop abdominal obesity during the 11-year follow-up (Valtonen et al., 2012). In addition, individuals with diabetes and MDD were found to have significantly higher CRP levels than non-diabetic patients with depression (Alvarez et al., 2013). A new epidemiological study showed that obesity is associated with greater depressive symptoms and that CRP concentrations were independently associated with depression scores in analyses fully adjusted for sociodemographic and background variables (Daly, 2013). Finally, another recent and large epidemiological study of men and women aged 20–100 years observed that increasing CRP levels were associated with increasing risk for psychological distress and depression (Wium-Andersen et al., 2013). Interestingly, increased CRP levels have been show to exacerbate injury-induced gliosis in the brain (Hsuchou et al., 2012b). It is intriguing to speculate whether or not potentiation of injury-induced gliosis by CRP is due in part to its actions to increase blood-brain barrier permeability to noxious stimuli (Hsuchou et al., 2012a). Decreasing the permeability of the BBB to free fatty acids, cytokines and/or immune cells are avenues through which abdominal obesity and dyslipidemia can encourage neuroinflammation and subsequent mood impairments.

## Summary and perspectives

Obesity is largely driven by excessive energy intake which is a product of both the amount and caloric density of food consumed. Agricultural, economic and cultural factors contribute to an abundance of energy-rich and palatable foods in many parts of the Western world and favor the selection of products containing saturated oils and refined sugars. The psychological dynamics of the consumer weigh heavily at the food selection level. Emotional reactions to food can largely affect our future intake and can propel overeating. What is becoming increasingly evident is that, in turn, the nutrients we consume can over time influence our mood and behavior either via direct CNS actions or by their impact on energy metabolism, endocrine function and immunity. Findings from several fronts tie the development of depressed mood in obese individuals to overeating, central adipose mass and associated metabolic disruptions (Figure 1). Recent research findings propose that the overconsumption of saturated fats is a potential factor underlying obesity-induced depression. In view of the effect saturated fats to bias central fat deposition and the association between saturated fat intake and cardiometabolic (Micha and Mozaffarian, 2009, 2010) and neurological diseases (Takechi et al., 2010; Kanoski and Davidson, 2011), the link between saturated fats and depression is not surprising. Thus, to further our understanding of the development of depression during obesity we can build upon a wealth of knowledge about the metabolic disturbances associated with central obesity, how these disturbances affect signaling mechanisms and, while less vast, our expanding understanding of how these signals modulate neural functions. Identifying the root causes of depression during obesity will greatly benefit from much more neurobiological and behavioral research of the mood-regulating CNS sites and mechanisms through which metabolic, inflammatory and nutrient signals act.

Endocrine abnormalities are common in visceral obesity. As discussed, various findings suggest that alterations in cortisol, leptin, adiponectin and resistin levels are associated with depression and/or that these hormones have CNS actions to affect mood, anxiety and depressive-like behavior. Another hormone well-implicated in overeating an obesity development is the gut peptide ghrelin, and several studies suggest that ghrelin plays a key role at the interface of homeostatic control and neural circuits involved in reward and stress (*for review see Abizaid article in this issue*). We did not review a role for ghrelin, however, because there is currently no evidence specifically linking this hormone to depression. On the other hand, neuropeptide systems that intricately interact with metabolic hormones have been linked to the pathophysiology of mood disorders (for review see Mathe et al., 2007). For example, apart from its orexigenic action, neuropeptide Y (NPY) was shown to reduce signs of anxiety when injected centrally (Heilig et al., 1989; Heilig, 1995). In line with these earlier studies, recent work demonstrates that NPY mRNA levels were decreased in hippocampal homogenates of a rat genetic model of depression (Melas et al., 2013). NPY is also reduced in the CSF of patients with depressive disorder (Heilig et al., 2004) and this is reversed upon antidepressant therapy (Nikisch et al., 2005). Yet other central signaling molecules with strong ties to both energy metabolism and emotions are the endocannabinoids. A role for endocannabinoid signaling via the CB1 receptor in anxiety and depressive symptoms is well established (Mangieri and Piomelli, 2007; Gorzalka and Hill, 2011). A recent study demonstrates the importance of endocannabinoid signaling in the amygdala in the anxiogenic effects of palatable food withdrawal (Mangieri and Piomelli, 2007; Gorzalka and Hill, 2011; McLaughlin and Gobbi, 2012; Mechoulam and Parker, 2013) and thus add to the growing evidence that endocannabinoids may be another link between obesity and mood disorders.

Endocrine dysfunction in central obesity typically coincides with a mild inflammatory state. Central adipose stores are a source of inflammatory cytokines secreted by local monocytes and macrophages, and the release of another inflammatory molecule, CRP, is upregulated during obesity and inflammation. Several findings demonstrate the pro-depressive effects of inflammatory signals whereas others studies are uncovering the deleterious impact of hypothalamic inflammation brought on by DIO. At present, the mechanisms that initiate and propagate neuroinflammation during high-fat feeding are not fully known. Moreover, it is not clear if limbic regions controlling emotions and mood also succumb to this “metabolic inflammation” during high-fat feeding. It also remains to be established if immune cells that are activated during obesity can infiltrate the brain as they do in other chronic inflammatory conditions and thereby contribute to the neuroinflammatory response. Apart from the involvement of humoral factors, one should also consider the action of metabolic signals on vagal efferents to the CNS. Cytokine stimulation of vagal nerves can trigger neuroinflammatory responses (Capuron and Miller, 2011) and therefore may prove to be an important pathway to diet-induced neuroinflammation. Establishing the direct impact of these metabolic and inflammatory signals to obesity-induced depression will not prove easy as they are often interconnected and temporally overlap. To surmount such difficulties controlled animal studies employing conditional and time-resolved loss- or gain-of-function strategies will be very valuable for determining the distinct involvement of each of these factors in the development of depressive behavior during high-fat feeding and obesity.

### Conflict of interest statement

The authors declare that the research was conducted in the absence of any commercial or financial relationships that could be construed as a potential conflict of interest.
